# Antifragility in Climbing: Determining Optimal Stress Loads for Athletic Performance Training

**DOI:** 10.3389/fpsyg.2020.00272

**Published:** 2020-03-11

**Authors:** Yannick Hill, Adam W. Kiefer, Paula L. Silva, Nico W. Van Yperen, Rob R. Meijer, Nina Fischer, Ruud J. R. Den Hartigh

**Affiliations:** ^1^Department of Psychology, University of Groningen, Groningen, Netherlands; ^2^Department of Exercise and Sport Science, The University of North Carolina at Chapel Hill, Chapel Hill, NC, United States; ^3^Center for Cognition, Action & Perception, Department of Psychology, University of Cincinnati, Cincinnati, OH, United States

**Keywords:** complex systems, hormesis, metastability, phenotypic plasticity, resilience

## Abstract

In the past decades, much research has examined the negative effects of stressors on the performance of athletes. However, according to evolutionary biology, organisms may exhibit growth under stress, a phenomenon called antifragility. For both coaches and their athletes, a key question is how to design training conditions to help athletes develop the kinds of physical, physiological, and behavioral adaptations underlying antifragility. An answer to this important question requires a better understanding of how individual athletes respond to stress or loads in the context of relevant sports tasks. In order to contribute to such understanding, the present study leverages a theoretical and methodological approach to generate individualized load–response profiles in the context of a climbing task. Climbers (*n* = 37) were asked to complete different bouldering (climbing) routes with increasing loading (i.e. difficulty). We quantified the behavioral responses of each individual athlete by mathematically combining two measures obtained for each route: (a) maximal performance (i.e. the percentage of the route that was completed) and (b) number of attempts required to achieve maximal performance. We mapped this composite response variable as a function of route difficulty. This procedure resulted in load–response curves that captured each athlete’s adaptability to stress, termed phenotypic plasticity (PP), specifically operationalized as the area under the generated curves. The results indicate individual load–response profiles (and by extension PP) for athletes who perform at similar maximum levels. We discuss how these profiles might be used by coaches to systematically select stress loads that may be ideally featured in performance training.

## Introduction

In competitive sports, athletes constantly interact with stressors, which represent events that athletes need to adapt to. Sport scientific research on stressors typically focuses on understanding and identifying strategies to promote athletes’ ability to return to their previous level of functioning following exposure to a stressor (Hill et al., 2018a, b). This ability, termed resilience, often presupposes a *negative* effect of stressors (Sarkar and Fletcher, 2013; Galli and Gonzalez, 2015). There is no question that stressors can disrupt the state of the athlete both on short timescales (e.g., losing a point) and long timescales (e.g., suffering an injury). However, previous research has shown that biological systems, under certain conditions, are capable of changing their structure and behavioral patterns when exposed to stress leading to growth rather than disruption in function (e.g., Cowin and Hegedus, 1976; Calabrese, 2005a). Growth from stress, termed antifragility (Taleb, 2012), is nicely illustrated when athletes implement novel and creative task solutions “on the fly” in response to challenges created by opponents in the field of play (Kiefer et al., 2018). Antifragility is ubiquitous in complex biological systems (Costantini et al., 2010; Calabrese and Mattson, 2011; Kiefer et al., 2018) and should therefore be a central target of sports training.

For both coaches and their athletes, a key question is how to design training conditions to help athletes develop the kinds of physical, physiological, psychological, and behavioral adaptations underlying resilience and antifragility. Research on psychological resilience shows that optimal adaptive responses to stressors are more common in individuals who have been exposed to intermediate loading in terms of lifetime adversity (Seery et al., 2010; Seery, 2011). Individuals who experienced either high or low amounts of stressors demonstrated lower levels of adaptability. Interestingly, such findings extend beyond psychological development and are in accordance with various stress–response processes studied in the field of evolutionary biology, medicine, toxicology, and sports (see for a review Costantini et al., 2010; Agathokleous et al., 2018). For example, human immune systems exhibit a response profile that is dependent on the toxicity (stress) that infectious agents impose to it: if the stress is too low, there is no response; if the stress is too high, it is harmful (Calabrese, 2005a). Vaccination is an effective treatment in that it imposes an optimal *level* of toxicity to “train” the immune system to respond to infectious agents. Similarly, following a (not too severe) bone fracture, the remodeling process of the bone produces tissue that is prepared to bear greater loads than before (Cowin and Hegedus, 1976). Also, following strength training with appropriate levels and types of load, muscle tissue grows (Jones et al., 1989) and is able to better respond to stress (Ocarino et al., 2008; Aquino et al., 2010). In the domain of sport psychology, clues for facilitative responses under specific loading can be derived from arousal–performance relationship theories (e.g., Kerr, 1985). Specifically, when athletes are somatically and cognitively under-aroused, increasing their level of arousal also increases their athletic performance until a threshold is exceeded and performance declines with increasing somatic and cognitive arousal (Hardy, 1990).

**FIGURE 1:**
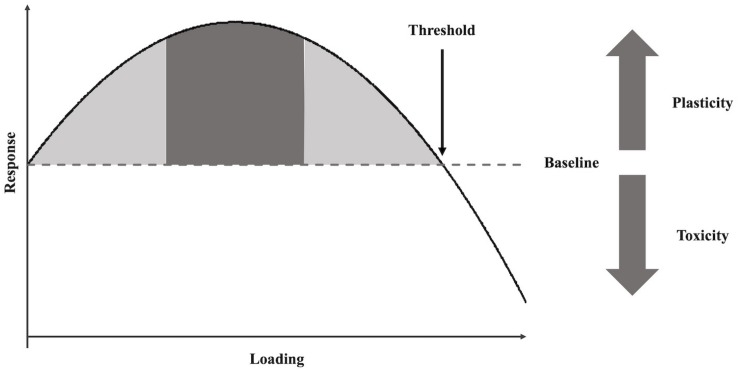
Hypothetical hormetic (i.e. biphasic) response curve for athletic performance training. The solid black line represents the system’s response to the increasing stress load relative to the system’s baseline (dashed line). The gray areas represent a system’s plasticity (or antifragility), while the dark gray area represents the maximum response of a system.

These examples capture a phenomenon, which can be observed across a broad range of biological systems, called *hormesis* (Southam and Ehrlich, 1943; Calabrese, 2005b; Costantini et al., 2010; Mattson and Calabrese, 2010; Agathokleous et al., 2018). Hormesis describes the biphasic relationship between the dosage of a potential harmful stressor and the response it triggers in an organism. Specifically, if the dosage is too small, it may yield a smaller beneficial effect in the immediate term; if the dosage is too large, it may trigger the opposite effect relative to baseline (Figure 1). Therefore, in order to elicit a desirable response, an optimal level of a stressor (or load) must be defined (Jaspers et al., 2018, 2019; Van der Sluis et al., 2019).

While useful for understanding dose–response dynamics in complex biological systems, the symmetrical shape and biphasic characteristics of the hormetic response curve illustrated in Figure 1 is not representative of all biological systems or organisms. For example, during strength training, the optimal load is known to differ between individuals (Jones et al., 1989). Just as biological organisms with similar genotypes express vastly different phenotypic responses to environmental extremes (Ghalambor et al., 2007; Costantini et al., 2010), athletes adapt and ultimately perform differently in the face of adversity. This means that two athletes, who perform at a similar level, may differ substantially in terms of how they adapt to different loading. For example, two athletes who can run a given distance in the same amount of time under low stress training conditions may perform very differently when environmental circumstances become more challenging due, for instance, to a temperature change. One athlete may need substantially more time with increasing heat, whereas another athlete may not differ very much from his or her personal best or even improve with increasing temperature (i.e. loading). Thus, it is necessary to individualize stress loads to trigger facilitative responses in the training context (Kiefer et al., 2018). To identify the optimal (training) load for each athlete, the hormetic curve can be used to quantify each athlete’s *phenotypic plasticity* (PP)—i.e. the athlete’s readiness to adapt to stress. PP can be quantified as the area under an athlete’s hormetic curve (Calabrese and Mattson, 2011; Kiefer et al., 2018). The resulting profile provides a systematic way to identify loads that can be expected to trigger optimal behavioral responses, those that might be too small to trigger beneficial responses, and those that might be too large for the system to maintain proper functioning. In the previous example of the runners, the time needed to cover the specified distance would be plotted as a function of increasing temperature to pinpoint under what temperature loading the optimal response of each athlete is triggered. Therefore, the response pattern that emerges from exposure to different levels of loading not only provides insight into the maximum performance level athletes can reach but also provide more nuanced yet relevant information about their adaptability to stress (or fitness).

The concepts of hormetic responses and PP have been successfully employed in the field of evolutionary biology for optimizing stress levels in a variety of biological systems (Costantini et al., 2010; Kiefer et al., 2018). However, they have yet to be applied to an athletic context (for a comprehensive review outlining the theoretical underpinnings of hormesis/PP in the context of athletic performance, see Kiefer et al., 2018). One barrier to application of this promising conceptual framework is the lack of objective measures to determine optimal loads for athletes in order to optimize performance development, enhance resilience, and promote antifragility. These objective measures are necessary to accurately map the changes in the response variables (e.g., running speed) as a function of loading (e.g., temperature). Equipping coaches and athletes with the necessary objective measures can help them design scientifically grounded training routines that facilitate athletes’ self-improvement in a safe training environment.

### The Current Study

The aims of the current study are (1) to provide a first empirical step toward the application of hormesis and PP to athletic performance training and (2) to determine whether the pattern of the hormetic response profile could be utilized to develop specific training recommendations. To achieve these aims, we designed a study involving a bouldering (climbing) task. In bouldering, loading can objectively be operationalized on the basis of the different difficulty degrees of particular routes (Draper, 2016). Although performance consists of many constituent variables, which could potentially be utilized for building load–response profiles, we assessed each athlete’s climbing performance in terms of the degree to which a route was completed as it provides an objective performance indicator inherent to each motor task (i.e. the route). Additionally, we recorded the number of attempts the athletes required to reach the maximum performance per route. These values were combined into a response variable. We mapped this composite response variable as a function of route difficulty. This procedure resulted in load–response curves that captured each athlete’s adaptability to stress, or PP, specifically operationalized as the area under the generated curves. Because similar genotypes demonstrate vastly different phenotypic expressions at loading extremes (Ghalambor et al., 2007; Costantini et al., 2010; Kiefer et al., 2018), Hypothesis 1 states that a group of climbers who reach similar maximum performance levels will exhibit a large range of PP scores (i.e. area under the load–response curve). Furthermore, across individuals, we expected to observe the typical characteristics of hormetic response curves, with evidence of antifragility. Specifically, Hypothesis 2 was that loading levels yield functional responses that intensify with increasing loading before reaching a peak amplitude. Following the peak amplitude, the response pattern begins to reverse until the athlete’s performance begins to degrade and they are ultimately unable to perform (Cowin and Hegedus, 1976; Calabrese, 2005a, b; Calabrese and Mattson, 2011).

Finally, we will discuss how the load–response profile can be utilized to develop specific training programs. Specifically, the anticipated profiles indicate under what loading athletes are not sufficiently challenged (i.e. easy routes, which are completed in a single attempt), under what loading the athlete’s capabilities are exceeded (i.e. unsuccessful completion regardless of the number of attempts), and when loadings trigger adaptive responses (i.e. completion of the routes in several attempts) (Kiefer et al., 2018). The identification of a systematic and objective strategy to assess how athletes respond to loading is a necessary step toward the development of training programs based on athlete- and task-specific PP (i.e. environmentally triggered, adaptive change).

## Materials and Methods

### Participants

We recruited 37 intermediate-level climbers (26 male, 11 female) who voluntarily signed up to participate in the study by distributing flyers at a bouldering gym and advertising the study on social media. Eligibility for participation required a climber to be able to, at minimum, successfully complete bouldering routes equivalent to the difficulty of 5A according to the French bouldering grade system (Draper, 2016), which is the classification of the easiest route in the current study. The mean age of the participants was 26.1 years (*SD* = 4.8), with one individual not disclosing his or her age, and a group average bouldering experience of 3.1 (*SD* = 2.7) years.

### Experimental Design and Setup

**FIGURE 2:**
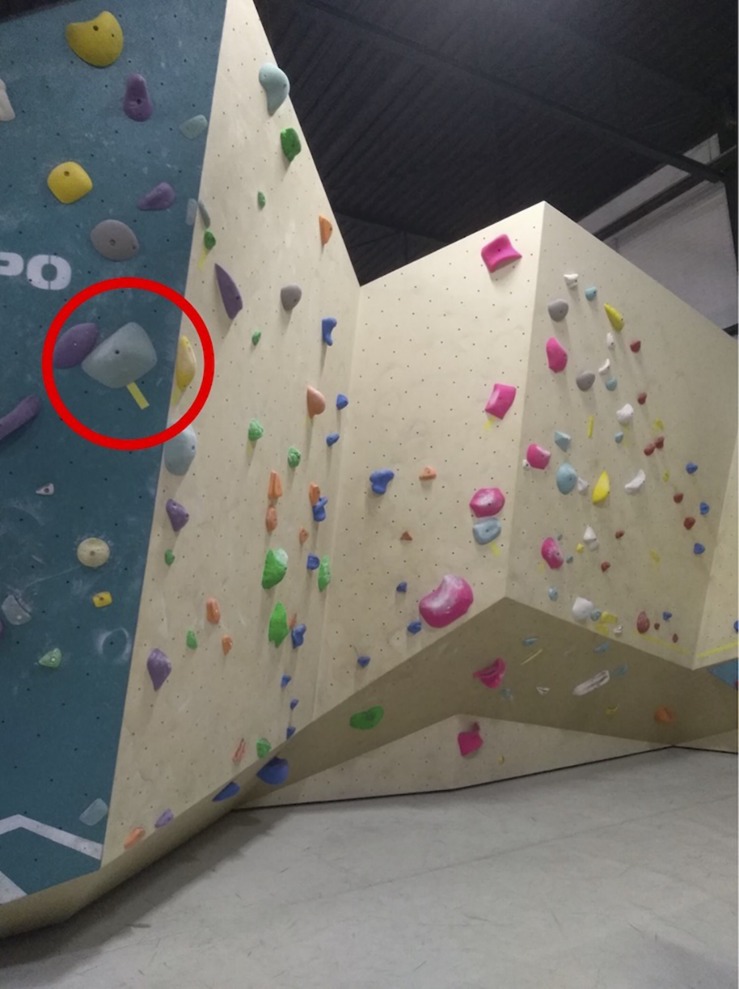
Photograph of the experimental setup with most of the included routes. The zone holds were marked with yellow stripes for the athletes’ clarity (an example is marked with the red circle). Consecutive holds of the same coloration yield one route.

The current study was conducted in a local bouldering gym. Eleven different bouldering routes were used in the current study and were designed by professional route setters to provide a proportional increase in difficulty from one route to the next, ranging from 5A (easy) to 7B (very difficult) according to the French grading system (Draper, 2016). The routes were designed to optimally support data collection. The wall was largely vertical with little overhang to ensure that athletes do not fail a route due to limited strength alone and to allow us to obtain clear video images with a straight angle. Furthermore, the holds and intended climbing technique were not systematically varied between routes by setters (in general, easier routes involved easier holds and leader-type climbing, which changes to increased finger strength and technical abilities with more difficult holds). The different routes were assigned specific color codes used in the gym to indicate the expected level of difficulty for the athletes. Therefore, we relied on the experts’ assessments of increasing difficulty in the rank order of the routes. Each route contained at least one zone hold, while three routes (i.e. route numbers 5, 10, and 11) contained two zone holds. A zone hold represents a marked hold on the route, which indicates partial route completion. Because the athletes’ performances were videotaped (using a GoProHero3+©, GoPro, Inc., United States) during both trials, all routes were placed at the same wall in order to (a) optimize the transitions between routes without requiring major changes to the setup and (b) minimize disruption to the flow of the athletic performance (see Figure 2).

### Procedure

The study procedure was approved by the Ethical Committee Psychology, University of Groningen (research code “18237-O”). Upon arrival at the bouldering facility, participants received information about the study and filled in the informed consent form. During the study, participants used their own equipment (e.g., climbing shoes and outfits). The warm-up program lasted approximately 20 min, and consisted of several body weight and stretching exercises as well as short bouldering on easy routes (grade 3, French grading system) in a different part of the facility. After the warm-up session, the actual data collection began. First, the participants climbed a maximum of 11 routes in a fixed order of increasing difficulty. The participants were instructed to complete as many routes as they could within the allotted time of 10 min. They were only allowed to move to the next more difficult route once a route had been completed. The number of attempts per route was not limited, and the athletes were encouraged to approach the routes as they would in regular training. For example, if they required more time to visualize a route before attempting it, they were allowed to do so. However, in order to avoid injuries by exposing athletes to overwhelming stress, the trial was terminated when a participant was unable to complete a given route (i.e. the participant could not reach the final hold and decided to stop or the 10 min had passed).

After the first trial, the participants sat at a desk with their backs facing the climbing routes and filled out a questionnaire assessing their demographics, physical fitness, and bouldering and climbing experience. During this 10–15-min break, the participants were also provided with refreshments and time to rest. However, during the break, the participants did not talk to other athletes in the facility and could only ask the experimenter questions related to the study. Furthermore, the participants were prohibited from seeing the routes and other athletes climbing these routes in order to avoid visualization effects (e.g., Sanchez et al., 2012; Orth et al., 2016). Following the break, the athletes conducted the second trial with the exact same routes in the same order. Afterward, the participants had the opportunity to receive a copy of the video files for both trials alongside a full debriefing of the study.

### Measures

#### Performance

For each route, the participants received a score that varied between 0 (i.e. not reaching a zone hold or the final hold) and 1 (i.e. successful completion of the route) for every attempt they conducted. We considered that an athlete successfully completed a route when the final hold was reached and held for 2 s, which was signaled by the experimenter. Reaching a zone hold yielded a proportional completion score depending on the number of zone holds per route (see Table 1 for possible scores). In line with the rules of the International Federation of Sport Climbing (International Federation of Sports Climbing [IFSC], 2019), we considered that an athlete reached a zone hold if he or she used the hold to produce a stable or controlled position or to progress along the route. Specifically, to gain the score for reaching a zone hold, the athlete had to: (a) make contact with the zone hold with one foot or hand while remaining in a stable position for at least 2 s, (b) use the zone hold to stabilize before progressing, or (c) use the zone hold to quickly progress with no interruption. Thus, shortly tapping the hold before falling onto the safety mats did not count as reaching the zone hold. Once an athlete was unable to successfully complete a route, the subsequent routes were also scored with 0.

**TABLE 1:** Possible scoring outcomes for performance for a given attempt.

Coding result	Completion rate	Performance
0 holds	0%	0
1 out of 2 zone holds	33.33%	1/3
1 out of 1 zone hold	50%	1/2
2 out of 2 zone holds	66.67%	2/3
Reaching final hold	100%	1

#### Attempts

The video footage was coded for the amount of attempts a participant required for each route. An attempt was counted if the athlete had both hands and feet on the starting holds and was thus off the ground. Any contact with the ground without successfully completing the route granted the opportunity to make a new attempt. There were no restrictions in the total number of attempts: the athletes were free to decide how many times they wished to attempt a given route.

### Data Analysis

The first step of the data analysis was to determine the maximum performance that each athlete achieved per route in each trial. For example, if a participant required more than one attempt but managed to complete the route, the maximum performance score reflected successful completion (i.e. a score of 1; Table 1). To assess systematic differences among trials, we computed the mean scores and the standard deviations of the number of attempts, the accumulated maximum performance scores for each route, and the number of routes completed for each trial. In order to account for potential learning effects and random variation, we averaged the maximum performance and the number of attempts per route of the trial before the break and the trial following the break. In order to assess each climber’s responses to loading (determined by a given route), we computed a “response” variable normalizing the average maximum performance by the average number of attempts:

(1) $$\text{Response}=\frac{M_{\text{Perf}}}{M_{\text{Att}}}$$

*M*_Perf_ equals the average maximum performance, whereas *M*_Att_ equals the average number of attempts. This equation yields values between a score of 1, reflecting route completion in a single attempt across both trials [i.e. *M*_Perf_ (= 1) divided by *M*_Att_ (= 1)], and 0 (i.e. no zone hold reached regardless of number of attempts across both trials). To illustrate, if a participant reached on average the second zone hold for a route in three attempts, they would earn a final response score of 0.222 (2/3 divided by 3, see Table 2 for an elaborate example and https://dataverse.harvard.edu/dataset.xhtml?persistentId=doi:10.7910/DVN/FJZ9AX for the full dataset). The resulting “response” scores were then plotted as a function of increasing loading (i.e. by increasing route difficulty) to create a load–response curve.

**TABLE 2:** Full response calculation example.

Route	Trial 1		Trial 2		Average		Calculation	Response
	Comp	Att	Comp	Att	Comp	Att	*M_Perf_/M_Att_*	
1	1	1	1	1	1	1	1/1	1
2	1	1	1	1	1	1	1/1	1
3	1	3	1	1	1	2	1/2	0.5
4	1	2	1	1	1	1.5	1/1.5	0.667
5	1	8	1	1	1	4.5	1/4.5	0.222
6	1	1	1	2	1	1.5	1/1.5	0.667
7	1	1	1	5	1	3	1/3	0.333
8	0.5	2	0	5	0.25	3.5	0.25/3.5	0.071
9	0	–	0	–				0
10	0	–	0	–				0
11	0	–	0	–				0

*Comp represents completion rate of the route for the trial, Att represents the number of attempts per route for the trial. The athlete does not manage to complete route 8 in each of the trials, which ended a given trial. The subsequent performance scores are set to 0.*

To quantify the range of PP among athletes, the analysis followed three steps. First, the area under the load–response curve was determined for each athlete. Because the loading on the *x*-axis represents discrete values with a constant loading interval (i.e. is an ordinal variable), the area under the curve can be approximated accurately by a cumulation of the response values on the *y*-axis:

(2) $$A\,U\,C=\sum_{i=1}^{n}R_{i}$$

*AUC* represents the area under the curve, *n* the maximum number of routes in the study (i.e. 11), and *R*_i_ the “Response” value at a given route. Hence, the example outlined in Table 2 would yield a PP (i.e. area under the curve) of 4.46 (given by 1 + 1 + 0.5 + 0.667 + 0.222 + 0.667 + 0.333 + 0.071).

Having determined the PP per individual (Step 1), we tested whether climbers who had reached similar maximum performance levels exhibit different PP scores (Hypothesis 1). Specifically, as a second step, the resulting PP scores were sorted according to the maximum performance the athlete reached (i.e. the most difficult route for which a response value larger than 0 on the *y*-axis was determined. In the example of Table 2, this would correspond to route number 8). Third, the maximum range of PP scores was then calculated for each group, with each group defined as two or more climbers who reached the same maximum performance. The maximum range scores were then averaged across all groups that contained at least two individuals. Because all athletes within each group reached the same maximum performance level, the mathematically maximum possible range is limited by the value of the maximum performance level of a given group. For example, for a group reaching a maximum performance of 7, the maximum range can be any value below 7, but not equal to 7. A range approximating a value of 1 is interpreted as large and represents the maximum response score an athlete can reach on a given route. In other words, a range equal to 1 within a group demonstrates that there is a difference of at least one optimal performance route despite reaching the same maximum performance.

To test for antifragile (i.e. growth from loading) properties of hormetic response curves (Hypothesis 2), we calculated whether positive deviations from the baseline value (i.e. the response score on the first route) precede negative deviations as a function of increasing loading. The hypothesis is supported if positive deviations from the baseline score precede negative deviations across participants.

## Results

**FIGURE 3:**
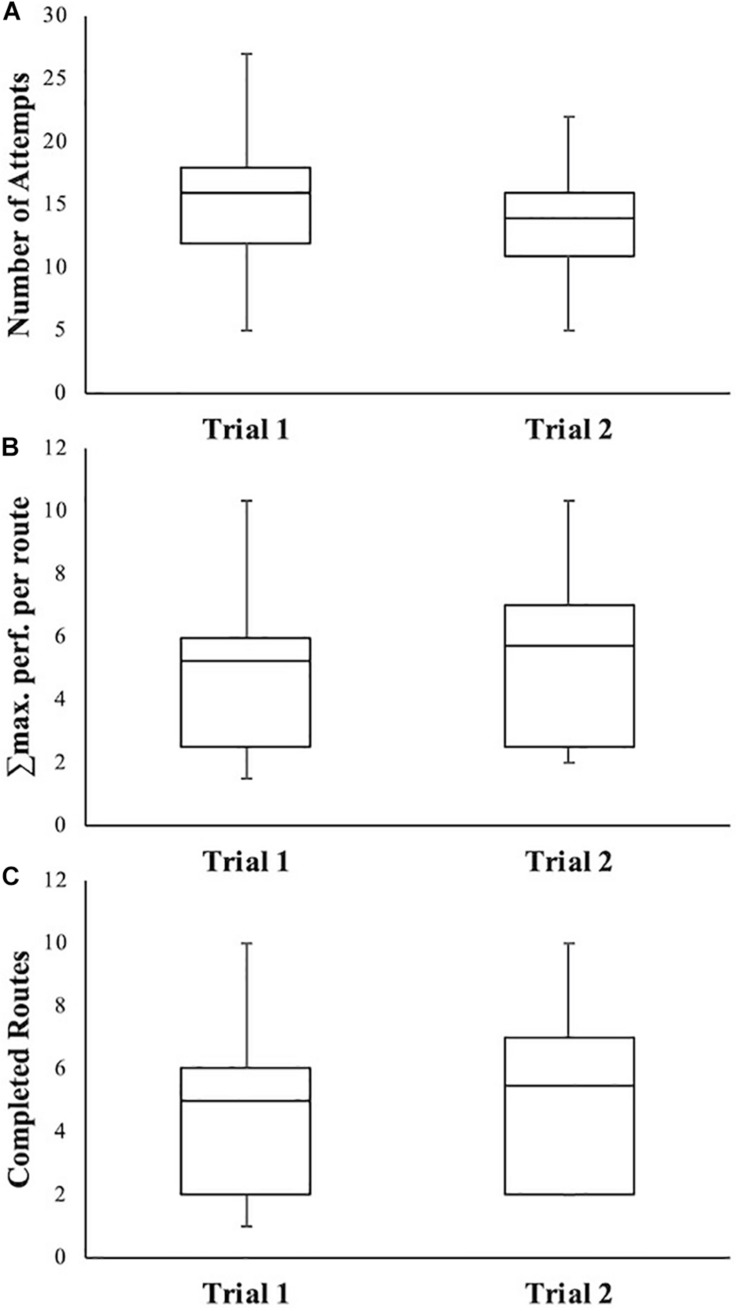
Boxplots depicting the medians, quartiles, minimum, and maximum of the number of attempts **(A)**, accumulated maximum performance **(B)**, and number of routes completed **(C)** for each trial.

Before conducting the main analyses, we assessed the differences between the trial before the break and the trial following the break. Athletes used on average 15.4 (*SD* = 4.5) attempts for the first trial and 13.8 (*SD* = 3.7) for the second trial. The maximum performance reached was, on average, 4.5 (*SD* = 2.2) on the first trial and 4.8 (*SD* = 2.5) on the second trial, while the average number of completed routes was 4.4 (*SD* = 2.3) for the first trial and 4.6 (*SD* = 2.5) for the second trial (see Figure 3 for a graphic illustration of the distributions). Thus, there seems to be a slight increase in maximum performance and number of routes completed, while the number of attempts between the two trials slightly decreases.

**FIGURE 4:**
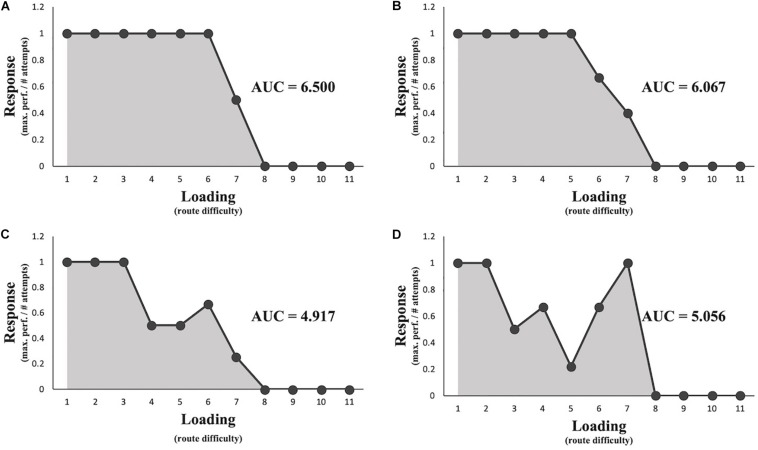
Four examples of different athletes’ load–response curves reaching the same maximum performance level **(A–D)**. The curves are created by mapping the response scores (see Eq. 1) as a function of loading determined by routes ordered from easiest (1) to most difficult (11). The gray area under the curve (AUC) score (i.e. the sum of an individual’s response scores, see Eq. 2) represents an athlete’s phenotypic plasticity (PP).

Hypothesis 1 was that athletes who reach similar maximum performance levels display a large range of PP. To test this hypothesis, we assessed the maximum range of PP within a group of athletes who reach the same maximum performance in terms of route completion. Grouping the athletes according to their maximum performance scores yielded eight different routes, where at least two individuals reached their maximum performance level (see Table 3 and supplementary material) with a mean average range of 0.951 (*SD* = 0.377). Because the average range approximates 1 (i.e. maximum response score for a given route), this provides an indication that athletes reaching the same maximum performance level indeed show considerably different adaptability under different loading extremes (Hypothesis 1). For example, Figure 4 represents four different athletes who complete the same number of routes but display unique load–response curves each and accordingly a large range of PP scores.

**TABLE 3:** Maximum range values of phenotypic plasticity for different maximum performance (Max. Perf.) by route.

Max. Perf. by route	*n*	Maximum range
2	6	1.083
3	7	1.258
4	6	0.833
5	5	0.967
6	3	0.929
7	4	1.583
8	1	0
9	2	0.583
10	2	0.373
11	1	0

*The size of each group is given by n.*

Hypothesis 2 was that the resulting profiles show typical properties of the hormetic response curve. That is, functional responses increase with increasing loading until a peak amplitude is reached, after which the pattern is reversed. Results obtained from the analysis of response profiles did not show the expected increase in performance from baseline with low levels of load before showing a decrease in performance with higher levels of lead. Thirty-six out of the 37 participants reached the maximum performance score (i.e. a score of 1) for the first route and therefore did not allow for any positive deviation from the baseline score for the subsequent routes (see Figure 3 for examples). Therefore, Hypothesis 2 is not supported.

## Discussion

The aim of the current study was to provide a first empirical step toward the application of hormesis and PP to human performance (Kiefer et al., 2018). This approach has the potential to provide researchers, coaches, and athletes alike with specific methods to objectively determine the optimal loading for athletic performance training. In order to test its feasibility in the context of sports performance, behavioral responses need to be initially examined as a function of increased loading (Costantini et al., 2010; Agathokleous et al., 2018; Kiefer et al., 2018). The resulting profiles can be analyzed to quantify an athlete’s PP by calculating the area under the load–response curve to determine the optimal training load for the athlete (cf. Calabrese and Mattson, 2011; Kiefer et al., 2018).

Our results suggest that load–response profiles provide novel information that can be used to generate specific recommendations for athletic performance training. That is, we found that athletes who reach similar maximum performance levels can demonstrate a rather large range of potential PP scores indicating their adaptability under various loadings. Due to this variability of load–response profiles (and by extension PP), any given profile is likely difficult to generalize to a broad range of athletes. Thus, the strategy must be personalized and starts with objective assessment of loading responses of each individual (Ghalambor et al., 2007; Costantini et al., 2010; Kiefer et al., 2018).

In line with the typical hormetic response curve, we expected the response curves of the athletes to first show an increase with intensifying loading before a critical point is reached after which the pattern reverses (e.g., Cowin and Hegedus, 1976; Calabrese, 2005a, b; Calabrese and Mattson, 2011). However, because all but one athlete reached the maximum response score on the first route, which served as the baseline for the fitness assessment, we did not observe enhancement in the behavioral response as a function of initial increases in loading. Thus, we cannot make inferences about antifragility in the observed athletes. The failure to find the expected pattern may be due to the fact that our baseline score does not represent the state of the athlete in the absence of any loading, as a true baseline score should (Costantini et al., 2010; Calabrese and Mattson, 2011). Since our response variable was a composite score of task-relevant behavior, it may not be possible to measure it in the absence of any loading. Future research should explore different measures, such as neuromuscular activity during performance, which allows measurements in the absence of loading.

Despite the absence of true antifragility evidence, the current load–response profiles of the athletes may still be utilized for training. Specifically, these profiles allow objective determination of routes that do not challenge the athlete, routes that exceed the capacity of the athlete, and routes that challenge the athletes. Routes that do not challenge the athlete are fully completed with a single attempt as they do not foster adaptations in the motor solutions employed to progress. When routes exceed the capacities of the athletes, they cannot make any progress (in terms of zone holds) independent of the number of attempts an athlete conducts. We classified routes situated in between these extremes as challenging because athletes can complete them but after more than one attempt. According to our scoring system, a response value of 1 would represent an easy route, a response score of 0 represents routes that exceed the capacity, and values ranging between 0 and 1 represent challenging routes: lower values indicate greater challenges. Challenging routes force the athlete to actively explore new motor solutions to adapt to his or her environment (Latash, 2012), which improves overall performance on routes of various difficulty levels (Seifert et al., 2014; Orth et al., 2016).

**FIGURE 5:**
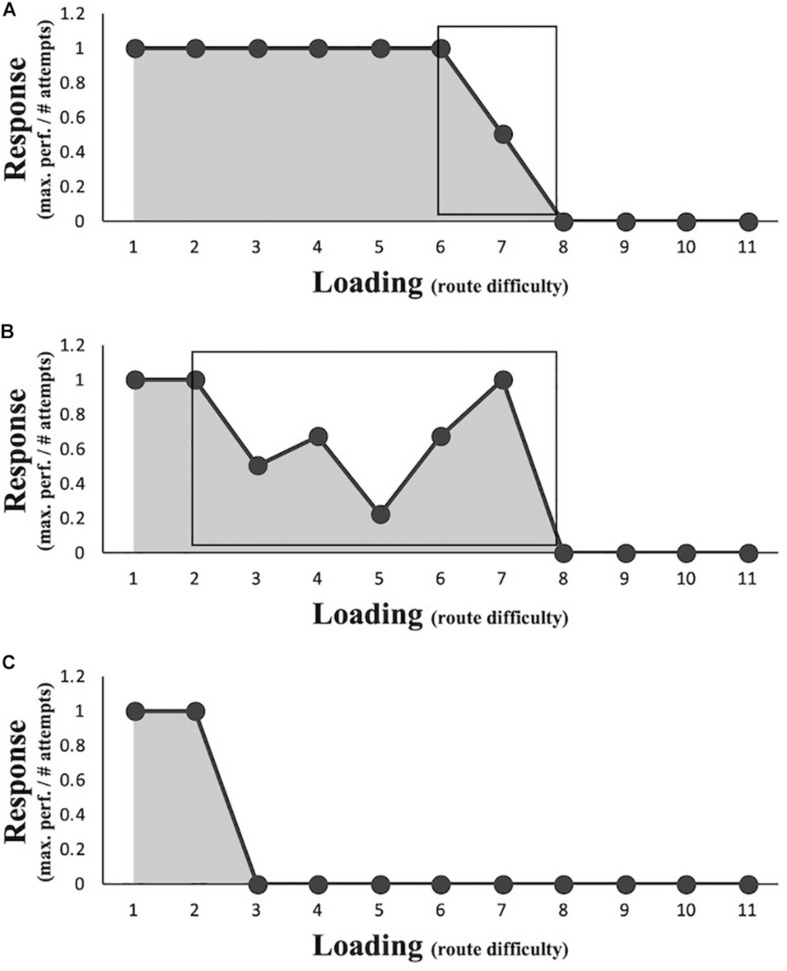
Three examples of load–response profiles for training recommendations. The black rectangles **(A,B)** represent the recommended training area residing between easy and too difficult routes. The athlete represented in **(C)** only demonstrates easy and difficult routes with no pattern in between.

In line with the variability of the response profiles (and therefore, PP) of the athletes, the range of challenging routes can differ between athletes, who reach a similar level of maximum performance (Figure 3; Kiefer et al., 2018). For example, most routes for the athlete displayed in Figure 4A may be considered too easy before the capacities are exceeded. This results in a rather small training window (Figure 5A). In contrast, the athlete displayed in Figure 4D encounters many challenging routes residing between too easy levels and exceedingly difficult levels, thus resulting in a relatively large recommended training window (Figure 5B). Creating load–response profiles can yield important insights into the stress–response of an athlete, which should be considered for his or her training regimes. In the current study, there was only one athlete who either easily completed a route or failed, leaving no “challenging” routes in the profile (Figure 5C).

The recommendations derived from the response profiles are also in line with research beyond the stress–response literature. For example, research on goal setting has shown that goals which are challenging but attainable yield optimal results in terms of performance and development (Locke and Latham, 2013; Van Yperen, 2020). Individuals setting easily attainable goals do not sufficiently challenge themselves, whereas individuals setting unattainable goals predispose themselves to failure, which can later be excused with the difficulty of the goals. In terms of hormesis, individuals who pursue challenging goals may expose themselves to loading that triggers positive behavioral responses but, due to the attainability of these goals, do not overload themselves.

### Limitations

In order to validate the specific recommendations for performance training derived from the load–response profiles, longitudinal studies need to be implemented. Specifically, studies identifying optimal loading for athletes should be coupled with designing training schedules leveraging this information. Athletes training under optimal loading should develop more motor solutions to behavioral task as well as improve their overall performance more than athletes training at suboptimal levels or who train based solely on information about maximal performance (i.e. conditioning hormesis; Calabrese et al., 2007). However, it should be noted that once the training routine begins, the optimal loading for athletes may change over time (as a function of changes in internal and/or external factors), thus requiring frequent monitoring of the plasticity profiles to ensure the exposure to optimal loading throughout such studies.

Although the current study provides an important first step toward the possible application of hormesis and PP to the domain of sports, our assessed response variables do not provide extensive information regarding the sport-specific behavior of the athletes. In order to fully translate the assessment of biological responses (i.e. response variables) to the domain of sport, in-depth measurements of the behavioral responses need to be obtained (Kiefer et al., 2018). For example, previous research has shown that non-linear complexity measures of athletic performance can provide insight into the dynamics of sport-specific movements (Den Hartigh et al., 2015; Kiefer and Myer, 2015; Araújo and Davids, 2016). Mapping such biological sport-specific variables as a function of loading may yield more sensitive and thorough profiles than overt behavioral measures. Such variables may also yield higher resolution for behavioral responses to variations in loading. In the current study, the maximum amount of data points that can be mapped as a function of loading may not be ideal for specific training recommendations as the patterns in the response profiles are based on discrete changes between two points rather than a trend of behavioral change of an athlete with successively increasing loading. Optimizing this resolution may increase the precision and effectiveness of specific recommendations derived from the response profiles (Calabrese et al., 2019).

Finally, in order to avoid injuries due to the exposure to loads that are too high, the athletes in the current study were asked to stop performing once they could no longer successfully complete a route. This also implies that the order of the routes had to be sorted by increasing difficulty and could not be completed in a randomized order. Fixing the order may have caused the athletes to become systematically more fatigued with increasingly difficult routes. Future studies may consider exposing athletes safely to increased levels of stress without risking harmful consequences by utilizing mixed reality [e.g., virtual reality (VR) or augmented reality] devices (Kiefer et al., 2017). Mixed reality environments may enable the safe exposure to highly standardized stressors while obtaining a multitude of sport-specific response variables in lab settings. Furthermore, securing safe exposure to varying stressors allows for a randomized presentation of different loadings. This, in turn, decreases the chance of finding lower response levels at higher levels of loading caused by fatigue. Ideally, virtual environments should be designed to capture the environmental information to which the athlete has to adjust during performance as closely as possible. This aspect of design is critical to optimize the chances that athletes will display responses to stressors in VR sports scenarios that are representative of those displayed in the real-world setting (Araújo et al., 2006).

### Implications

Establishing load–response profiles to optimize athletic performance training is not restricted to individual sports, such as climbing. It can be extended to other domains. For example, when different athletes perform together, they form a dynamical, biological system of constantly interacting individuals (e.g., Gorman et al., 2017). Thus, a sports team could be viewed as a system, which follows many of the same dynamic principles as individual athletes. Similarly, as evolutionary biology demonstrates, the notion of hormesis can be extended to a collection of organisms within the same species and colony (Mattson and Calabrese, 2010). Successfully adapting to small environmental hazards increases the biological fitness of a species, which increases the resistance to higher dosages of environmental hazards. This implies that load–response profiles can also be established for sports teams to pinpoint the optimal loading for performance training. In the case of crew rowing, for instance, there are several factors, such as coordination of the strokes of the individuals (e.g., Hill, 2002; Den Hartigh et al., 2014; De Poel et al., 2016), that contribute to the team’s performance and could thus be used as a response variable. Loading could be varied systematically by asking the teams to row a certain distance at different amounts of time (i.e. speed). The resulting profiles would then map coordination (i.e. response) as a function of speed (i.e. loading) to pinpoint at which speeds the athletes coordinate well or struggle to coordinate. Therefore, to extend load–response profiles to different sports, it is essential to define one or multiple response variables, which can be measured as a function of systematically varied loading.

For more dynamic team sports, such as soccer, it is much more difficult to identify a single determinant of player performance. Instead, it is likely more useful to obtain information from different load–response profiles for individual skills and behaviors to determine optimal loading. For example, avoiding collisions on the field can be regarded an important skill for a soccer player because it increases the chance of passing an opponent while simultaneously decreasing the chance of acquiring injuries (Silva, 2017). Using mixed reality devices, an athlete could be asked to complete a short sprint while trying to avoid virtual obstacles. Loading could be manipulated by varying the amount and difficulty (e.g., size and movement) of the obstacles. Then, the time the athlete needs to complete the sprinting route and the number of obstacles avoided can be combined with the response, which is plotted as a function of loading. Similar profiles can then be established for passing accuracy given the distance to the teammate and opposing players similar to game-relevant behaviors (Silva, 2017; Kiefer et al., 2018).

In addition to a quantitative approach, qualitative accounts may help explain the idiosyncratic shape(s) of load–response profiles. More specifically, interviews following the experiment or asking participants to verbalize their thoughts during the performance may help to match the specific strategies applied by the athletes to their load–response. This could help coaches to facilitate effective strategies.

## Conclusion

In conclusion, the current study provides a first empirical insight into the applicability of hormesis and PP to the assessment of athletic performance. We assessed climbers’ performance as a function of increasing difficulty in bouldering routes (i.e. loading). Our results suggest that the application of PP to assessment of adaptability to loading is scalable to human performance. Therefore, training programs that enhance both athletic performance and athletes’ adaptability to stressors (i.e. resilience, antifragility) should consider the load–response curves of individual athletes for a more precise and personalized intervention. These profiles enable researchers and coaches to objectively determine optimal loading and provide a basis for understanding the resulting dose–response dynamics throughout athletic performance training.

## Data Availability Statement

The data that support the findings of this study are openly available in Dataverse at 10.7910/DVN/FJZ9AX.

## Ethics Statement

The studies involving human participants were reviewed and approved by the Ethical Committee of Psychology (ECP) RuG (University of Groningen). The patients/participants provided their written informed consent to participate in this study.

## Author Contributions

YH developed the theoretical framework, conceived the study, analyzed the data, and wrote the manuscript. AK and PS developed the theoretical framework, analyzed the data, and provided critical feedback on the drafts. NV conceived the study, guided the analyses, and provided critical feedback on the drafts. RM conceived the study and provided critical feedback on the drafts. NF conceived the study, collected the data, and aided the analysis process. RD developed the theoretical framework, conceived the study, guided the analyses, and provided critical feedback on the drafts.

## Conflict of Interest

The authors declare that the research was conducted in the absence of any commercial or financial relationships that could be construed as a potential conflict of interest.
